# Efficacy decrease of antiviral agents when administered to ongoing hepatitis C virus infections in cell culture

**DOI:** 10.3389/fmicb.2022.960676

**Published:** 2022-08-03

**Authors:** Carlos García-Crespo, Lucía Vázquez-Sirvent, Pilar Somovilla, María Eugenia Soria, Isabel Gallego, Ana Isabel de Ávila, Brenda Martínez-González, Antoni Durán-Pastor, Esteban Domingo, Celia Perales

**Affiliations:** ^1^Centro de Biología Molecular “Severo Ochoa” (CSIC-UAM), Consejo Superior de Investigaciones Científicas (CSIC), Madrid, Spain; ^2^Centro de Investigación Biomédica en Red de Enfermedades Hepáticas y Digestivas (CIBERehd) del Instituto de Salud Carlos III, Madrid, Spain; ^3^Department of Clinical Microbiology, IIS-Fundación Jiménez Díaz, UAM. Av. Reyes Católicos, Madrid, Spain; ^4^Departamento de Biología Molecular, Universidad Autónoma de Madrid, Madrid, Spain; ^5^Department of Molecular and Cell Biology, Centro Nacional de Biotecnología (CNB-CSIC), Consejo Superior de Investigaciones Científicas (CSIC), Madrid, Spain

**Keywords:** daclatasvir, sofosbuvir, favipiravir, ribavirin, direct acting antivirals, viral fitness, delayed drug administration, lethal mutagenesis

## Abstract

We report a quantification of the decrease of effectiveness of antiviral agents directed to hepatitis C virus, when the agents are added during an ongoing infection in cell culture vs. when they are added at the beginning of the infection. Major determinants of the decrease of inhibitory activity are the time post-infection of inhibitor administration and viral replicative fitness. The efficacy decrease has been documented with antiviral assays involving the combination of the direct-acting antiviral agents, daclatasvir and sofosbuvir, and with the combination of the lethal mutagens, favipiravir and ribavirin. The results suggest that strict antiviral effectiveness assays in preclinical trials may involve the use of high fitness viral populations and the delayed administration of the agents, relative to infection onset.

## Introduction

The intra-host population dynamics of RNA viruses can influence the efficacy of antiviral treatments by providing dynamic mutant spectra in which some genomes may encode amino acids that confer resistance to antiviral agents. Resistance-associated substitutions (RASs) may preexist in viral populations prior to treatment administration, or they may be generated and selected during replication in the presence of the corresponding antiviral agent. RASs are generally specific for an antiviral drug, and they have been described for most viruses for which antiviral treatments have been investigated or implemented. The presence of RAS at a sufficient frequency to permit virus escape depends on interconnected sets of factors such as the genetic barrier (number and types of mutations required to produce a RAS), the phenotypic barrier (the fitness cost inflicted by RAS), the error rate of the virus during replication, the distance of the population from a clonal origin, and the viral population size [examples and reviews of the effect of such multiple factors can be found in (Richman, 1996; Ribeiro et al., 1998; Ribeiro and Bonhoeffer, 2000; Domingo and Perales, 2012; Perales et al., 2017; Perales, 2018; Li and Chung, 2019; Domingo, 2020)].

The administration of direct-acting antiviral (DAA) agents has been highly successful for the control of chronic hepatitis C virus (HCV) infections, with sustained virological responses of around 95% (Janjua et al., 2021). Selection of RAS is responsible for a large proportion of HCV treatment failures (Di Maio et al., 2017, 2021; Kai et al., 2017; Ceccherini-Silberstein et al., 2018; Dietz et al., 2018; Perpinan et al., 2018; Sorbo et al., 2018; Lombardi et al., 2019; Chen et al., 2020; Malandris et al., 2021; Sarrazin, 2021). RASs have been identified in treatment-naive patients (Costantino et al., 2015; Kai et al., 2017; Li et al., 2017a,b; Esposito et al., 2018; Perales et al., 2018; Yang et al., 2018; Morishita et al., 2020), reflecting the impact of the basal HCV mutation rate and viral population dynamics on the response to antiviral agents.

An alternative mechanism of antiviral resistance was identified in HCV from chronically infected patients. It was revealed by the presence of a class of highly represented amino acid substitutions (HRSs) in the basal viral samples (before treatment onset) of patients who then failed therapy; HRSs were associated with resistance to several DAA treatments, comprising double and triple combinations that included sofosbuvir (isopropyl (2S)-2-[[[(2R,3R,4R,5R)-5-(2,4-dioxopyrimidin-1-yl)-4-fluoro-3-hydroxy-4-methyl-tetrahydrofuran-2-yl]methoxy-phenoxy-phosphoryl]amino]propanoate) and ribavirin (1-β-D-ribofuranosyl-1-*H*-1,2,4-triazole-3-carboxamide) (Soria et al., 2020). This RAS-independent mechanism may account for a sizeable proportion of HCV treatment failures that have been reported in several patient cohorts (Kim et al., 2014; Nakamoto et al., 2014; Mawatari et al., 2018; Uchida et al., 2018; Bellocchi et al., 2019). The molecular mechanism of HRS-mediated antiviral resistance is not known but it may relate to the effect of HCV replicative fitness on antiviral resistance. Antiviral resistance conferred by high viral fitness was revealed in studies with HCV in cell culture, and HRS may reflect a similar fitness effect *in vivo*, although this proposal needs to be proven. The high HCV fitness-mediated resistance to anti-HCV inhibitors in cell culture was documented with disparate classes of antiviral agents. The studies involved measurements with DAAs (sofosbuvir, telaprevir, or daclatasvir), interferon-α (IFN-α), cyclosporine A—which targets the cellular cyclophilin A—lethal mutagens such as the nucleoside analogs favipiravir (T-705; 6-fluoro-3-hydroxy-2-pirazinecarboxamide) and ribavirin [(Sheldon et al., 2014; Gallego et al., 2016, 2018), or the metabolite inhibitor guanosine (Sabariegos et al., 2022); reviewed in (Domingo et al., 2019)].

As a consequence of these observations, we became interested in antiviral designs that can be effective in inhibiting high fitness HCV. In this line, we described that favipiravir and ribavirin exert a synergistic antiviral effect on HCV during replication on human hepatoma Huh-7.5 cells (Gallego et al., 2019). Importantly, combinations of these two analogs extinguished high fitness HCV that was resistant to equivalent total doses of one of the analogs. Synergy may be due to differences in the mechanism of antiviral activity between favipiravir and ribavirin (Furuta et al., 2009; Beaucourt and Vignuzzi, 2014; De Clercq and Li, 2016) in addition to their lethal mutagenesis (error rate enhancing) activity during RNA synthesis. A difference in the preference for the genomic sites mutated by the two analogs—as revealed by deep sequencing—may have contributed to synergy (Gallego et al., 2019).

A critical question that has not been addressed in preclinical experiments with HCV is the effect on antiviral efficacy of the time of addition of the antiviral agents, relative to the time of initiation of the infection. Classic findings on the treatment of microbial infections in general, and in the use of antiretroviral agents for AIDS, indicated benefits of the implementation of treatments early after infection (Ehrlich, 1913; Ho, 1995). These observations fit predictions of models of viral dynamics (Nowak and May, 2000; Hadjichrysanthou et al., 2016). One of the models that integrated virological and immunological data in a system consisting of cynomolgus macaques infected with Ebola virus indicated that, to be effective, antiviral treatments with favipiravir and remdesivir (GS-5734) had to be administered at least 2 days before the peak of viremia and cytokine storm (Madelain et al., 2018). Similar predictions of the advantage of early antiviral administration were made using models of monotherapy to block SARS-CoV-2 using infection parameters from patients (Goncalves et al., 2020). The potential benefit of favipiravir to treat arenavirus infections (Mendenhall et al., 2011a) was reinforced by the observation that favipiravir was effective in guinea pigs infected with Pichinde virus, even when the drug was administered when the animals were ill (Mendenhall et al., 2011b).

The HCV-Huh-7.5 cell culture system—with the availability of HCV populations displaying different fitness (Sheldon et al., 2014; Moreno et al., 2017; Gallego et al., 2020; Delgado et al., 2021)—offered a unique opportunity to quantify the consequences for antiviral efficacy of adding DAAs and lethal mutagens once the infection has been already initiated. With this aim, we used two related HCV populations that belong to the same evolutionary lineage in Huh-7.5 cells in cell culture, but that differed in fitness. The low fitness HCV was the clonal population HCV p0, obtained by the transcription of plasmid Jc1FLAG2(p7-nsGluc2A) (Marukian et al., 2008), cell transfection with the RNA transcripts, and virus amplification in Huh-7.5 cells (Perales et al., 2013). HCV p0 was arbitrarily given the fitness value of 1. It was subjected to 200 serial passages in Huh-7.5 cells at a multiplicity of infection (MOI) of 0.03 TCID_50_/cell to yield population HCV p200 which displayed a fitness 2.3-fold higher than that of HCV p0 (Gallego et al., 2020). We report the consequences of adding DAAs [combinations of daclatasvir (dimethyl N,N'-([1,1'-biphenyl]-4,4'-diylbis{1H-imidazole-5,2-diyl-[(2S)-pyrrolidine-2,1-diyl][(2S)-3-methyl-1-oxobutane-1,2-diyl]}) dicarbamate) and sofosbuvir] or combinations of lethal mutagens, favipiravir and ribavirin, once the infection of Huh-7.5 cells by HCV p0 or its high fitness derivative HCV p200 is ongoing. Delaying inhibitor addition relative to initiation of the infection reduced significantly the antiviral efficacy, and the reduction was accentuated with the DAAs when cells were infected with the high fitness HCV population. We discuss the results in terms of replicative parameters and fitness effects on antiviral sensitivity. We suggest that preclinical protocols for the evaluation of antiviral agents would benefit from including the use of high fitness viral populations and tests of addition of the agents once the infection is well underway.

## Materials and methods

### Cells and viruses

Huh-7.5 reporter cells were used for all HCV infections, and Huh-7.5 cells were used for virus titration. Cells were grown in Dulbecco's modification of Eagle's medium (DMEM) at 37°C in a 5% CO_2_ atmosphere, following previously described procedures (Blight et al., 2002; Jones et al., 2010; Gallego et al., 2018). Cells were passaged a maximum of 30 times, using a split ratio of 1:4 before their use in the experiments.

The initial HCV population was obtained following virus rescue by the expression of plasmid Jc1FLAG2(p7-nsGluc2A) (a chimera of J6 and JFH-1 from HCV serotype 2a) (Marukian et al., 2008) and subsequent expansions in Lunet and Huh-7.5 human hepatoma cells to obtain HCV p0, as previously described (Perales et al., 2013). Likewise, a replication-defective HCV GNN [that includes a mutation in NS5B that abolishes polymerase activity (Marukian et al., 2008)] was rescued and used as negative infection control. HCV was passaged 200 times in Huh-7.5 cells to produce HCV p200, as previously described (Sheldon et al., 2014; Moreno et al., 2017). To control the absence of contamination, mock-infected and HCV GNN-infected cells were handled in parallel, and their supernatants were titrated; no evidence of contamination was obtained in any of the experiments.

### Antiviral agents and antiviral protocols

Stock solutions of daclatasvir [10 mM in dimethyl sulfoxide (DMSO); Selleck Chemicals], sofosbuvir (10 mM in DMSO; Selleck Chemicals), favipiravir (20 mM in water; Atomax Chemicals Co. Ltd.), and ribavirin (100 mM in PBS; Sigma) were prepared, sterilized by filtration, and stored at−70°C, as detailed previously (Sheldon et al., 2014; Gallego et al., 2016, 2018). Drugs were diluted in DMEM before their use to reach the desired concentrations for the experiments. In all experiments, 4 x 10^5^ Huh-7.5 reporter cells were infected with either HCV p0 or HCV p200 at a multiplicity of infection (MOI) of 0.03 TCID_50_/cell. After 5 h of virus adsorption to cells, the inoculum was removed, cells were washed, and fresh medium without or with the antiviral compounds was added. In different protocols, the time of addition of antiviral compounds relative to the initiation of infection and the duration of the infection varied, as indicated for each experiment. For serial viral passages, 0.5 ml of the cell culture supernatant from the previous infection was used to infect 4 x 10^5^ Huh-7.5 reporter cells. The infection continued in the absence or presence of the drugs for 72 to 96 h, as indicated in the relevant experiment.

### HCV titration and test of HCV extinction

For titration of infectious HCV, serial dilutions of the cell culture supernatants were applied to Huh-7.5 cells that had been seeded 16 h earlier in 96-well plates at 6,400 cells/well. Three days post-infection, the monolayers were washed with PBS, fixed with ice-cold methanol, and stained with NS5A-specific monoclonal antibody 9E10, as previously described (Lindenbach et al., 2005; Perales et al., 2013). Viral titers are expressed as 50% tissue culture infective dose (TCID_50_/ml) (Reed and Muench, 1938).

When no infectivity was detected, the cell culture supernatant was analyzed by the HCV extinction test (de Avila et al., 2016). It consists in subjecting the undiluted cell culture supernatant to three blind passages in Huh-7.5 reporter cells, in the absence of any drug; when no infectivity was detected in the cell culture medium of the third blind passage and no RT-PCR amplifiable material was detected in the intracellular material, the virus was considered extinct. The oligonucleotide primers used for the RT-PCR NS5A-F1 and NS5A-R1 are included in Supplementary Table S1.

### Quantification of HCV RNA

Total cellular RNA was extracted from infected cells or cell culture supernatants using QIAamp Viral RNA kit (Qiagen), according to the manufacturer's instructions; for intracellular RNA, the Qiagen RNeasy kit (Qiagen) was used. HCV RNA was quantified by real-time quantitative RT-PCR (qRT-PCR) using the LightCycler RNA Master SYBR Green kit (Roche) (Perales et al., 2013). A fragment of the 5' untranslated region (UTR) was used for the quantification; the oligonucleotide primers used for the amplification, termed HCV-5UTR-F2 and HCV-5UTR-R2, are included in Supplementary Table S1. Quantification was relative to a standard curve obtained with known amounts of HCV RNA synthesized by *in vitro* transcription of plasmid GNNFLAG2(p7-nsGluc2A). To ascertain the absence of contamination with undesired templates, negative controls (consisting of samples without added template RNA or including RNA from mock-infected cells) were run in parallel. This procedure and primers for HCV RNA quantification have been previously used (Perales et al., 2013; de Avila et al., 2016).

### PCR amplification and Sanger sequencing

RT-PCR amplification was carried out using AccuScript (Agilent) following the manufacturer's instructions. Amplification products were analyzed by agarose gel electrophoresis, using GeneRuler 1 kb Plus DNA Ladder (Thermo Scientific) as molar mass standard. Amplification controls in the absence of RNA were run in parallel to ascertain the absence of contamination by undesired templates. Amplified DNA was sequenced using the 23 ABI 3730 XLS sequencer (Macrogen, Inc.). All the oligonucleotide primers used in this study are listed in Supplementary Table S1.

### Statistics

The statistical significance of differences in infectivity and RNA levels was determined using the t-test and software GraphPad Prism 8.00. The differences between viral titers and RNA levels along the different passages were determined using ANCOVA test and software GraphPad Prism 8.00.

## Results

### Kinetics of hepatitis C progeny production and time-dependent decrease of antiviral efficacy

We previously determined CC_50_ for Huh-7.5 cells and IC_50_ values for inhibition of HCV progeny production for the DAAs, daclatasvir and sofosbuvir, and for the mutagenic nucleoside analogs, favipiravir and ribavirin (Table 1); these are the four inhibitors used in this study. Upon infection of Huh-7.5 cells, the progeny production of both HCV p0 and HCV p200 increased exponentially up to 72 h post-infection (Moreno et al., 2017); this result was confirmed in this study, with the additional note that the extracellular infectivity was maintained up to 144 h post-infection (Supplementary Figure S1).

**Table 1:** CC_50_ for Huh-7.5 cells and IC_50_ values for inhibition of HCV progeny production by antiviral agents.

**Antiviral agent**	**CC_50_**	**IC_50_**
Daclatasvir^a^	14,900 ± 600 nM	10 ± 0.3 pM
Sofosbuvir^b^	>50 μM	20 ± 3 nM
Ribavirin^a^	108 ± 4.2 μM	6.9 ± 0.9 μM
Favipiravir^c^	865 ± 59 μM	7.4 ± 6 μM

a *Data from Sheldon et al., 2014*.

b *Data from Gallego et al., 2016*.

c *Data from de Avila et al., 2016*.

**Figure 1:**
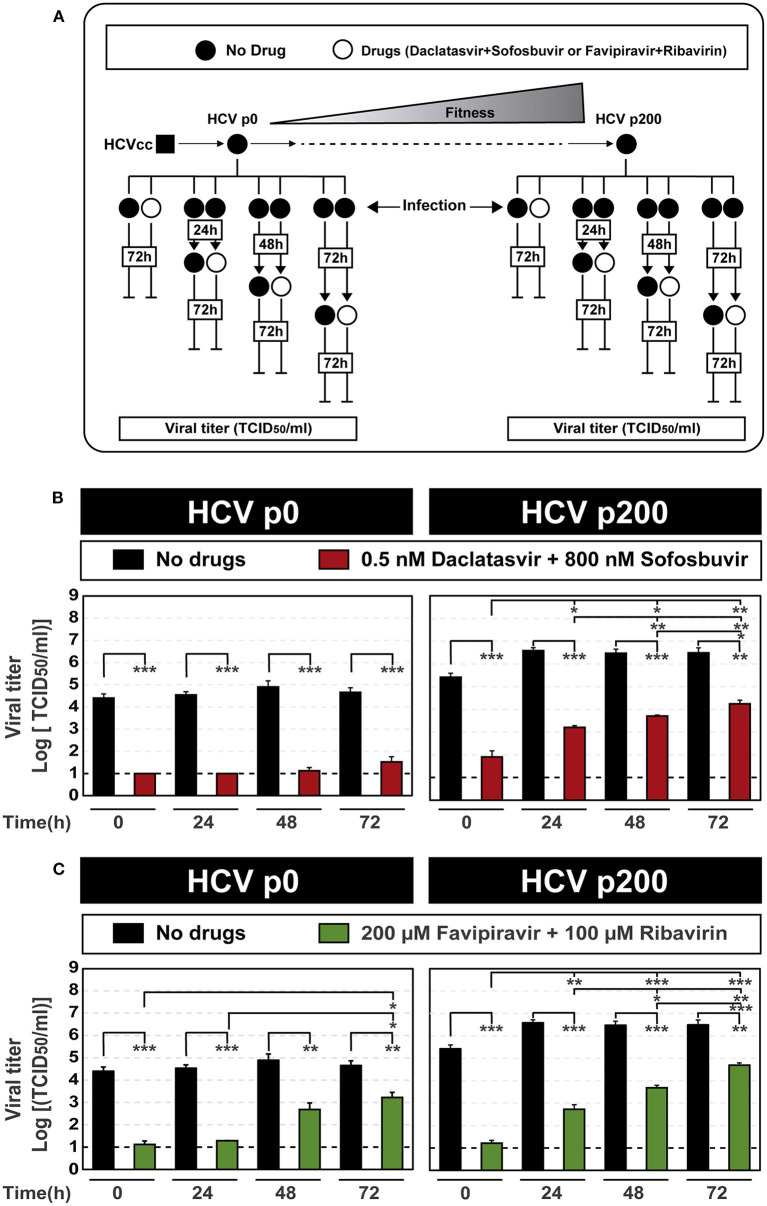
Effect of the time of addition of inhibitor combinations after initiation of HCV infection. **(A)** Scheme of the experiment, displaying fitness gain upon subjecting HCV p0 [derived from HCVcc obtained by transfection of Lunet cells with RNA transcribed from plasmid Jc1FLAG2(p7-nsGluc2A)] to 200 serial passages in Huh-7.5 reporter cells to yield HCV p200. The time of infection and of addition of the inhibitors are indicated (empty boxes inserted in vertical lines); in all cases, cell culture supernatants were titrated for HCV infectivity at 72 h following the last addition of inhibitors. **(B)** Virus titer upon the addition of combinations of the DAAs daclatasvir and sofosbuvir. The virus used for infection is indicated in the upper filled boxes, and the inhibitor concentrations in the cell culture medium are given in the empty box. The abscissa shows the time post-infection of addition of the drug combination. **(C)** Same as **(B)** except that the inhibitors used were combinations of the mutagenic nucleoside analogs favipiravir and ribavirin. For **(B,C)**, the statistical significance of the differences between values given in the bars are given as follows: *p < 0.05; **p < 0.01; ***p < 0.001; t-test). Viral titer values can be found in Supplementary Table S2. Extracellular RNA values can be found in Supplementary Table S3 and Supplementary Figure S2. Experiments were performed in triplicate (replicas A, B, and C in Supplemental Tables S2, S3). Procedures followed for drug preparation, cell infections, and titration of infectivity are detailed in Materials and Methods.

Based on the kinetics of progeny production, and the CC_50_ and IC_50_ values for the inhibitors used in this study, we evaluated the influence of the time elapsed between infection initiation and inhibitor addition on the efficacy of inhibitor combinations. With this aim, DAAs and lethal mutagen combinations were added at 0 h, 24 h, 48 h, and 72 h after infection, and viral titer was determined at 72 h after the addition of the inhibitors. The results (Figure 1) documented a decrease of antiviral efficacy that was more intense the longer was the infection time prior to inhibitor addition. The effect was more accentuated in the infections with HCV p200 than with HCV p0, particularly for the DAA combination (compare Figures 1B,C; the numerical values are given in Supplementary Table S2). The inhibitions were confirmed with quantifications of extracellular viral RNA (Supplementary Table S3; Supplementary Figure S2).

**Figure 2:**
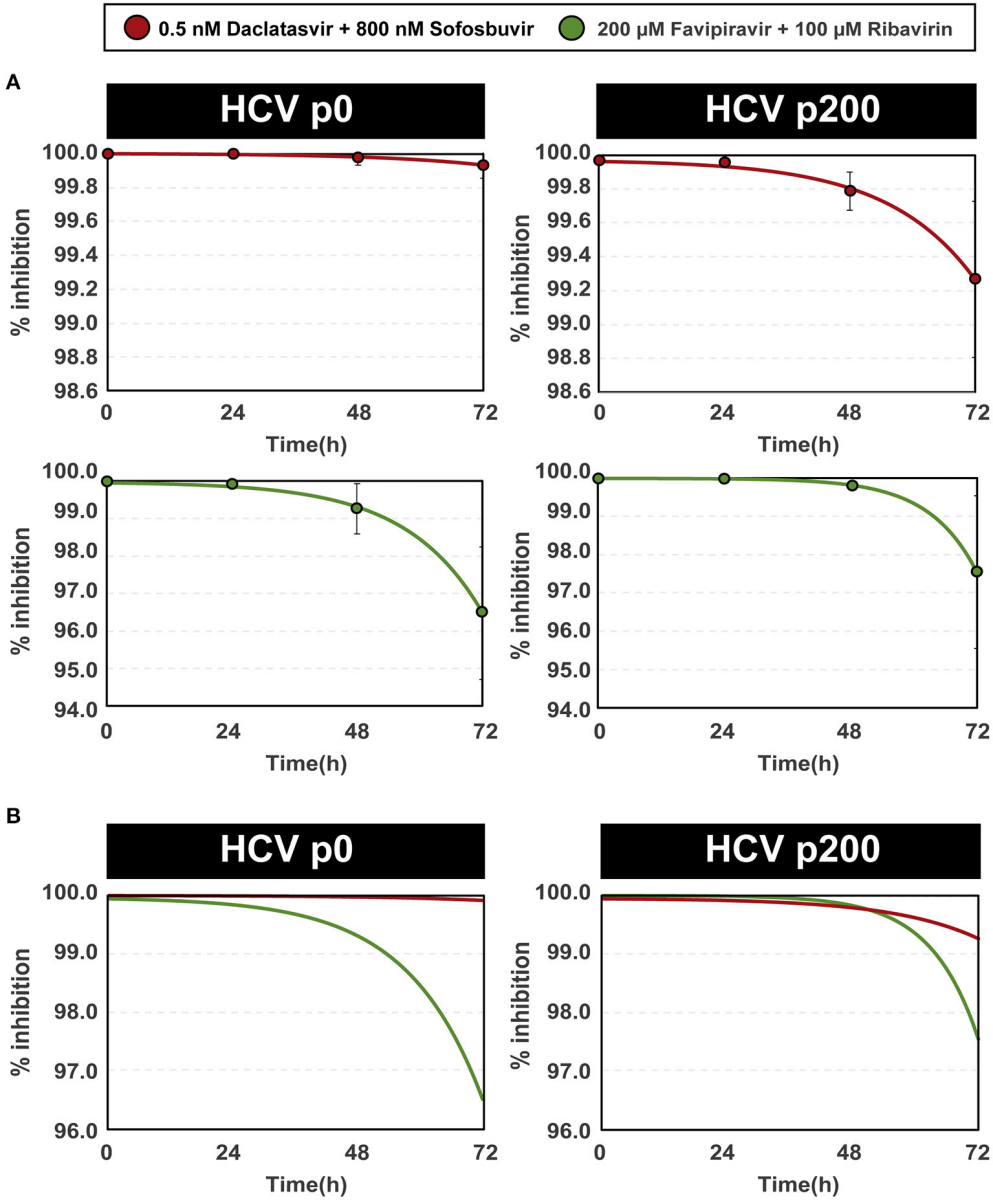
Percentage of inhibition of HCV progeny production as a function of the time of inhibitor addition after initiation of infection. **(A)** The percentages of inhibition have been calculated with the data given in Figure 1 and Supplemental Table S2. The inhibitors (color coded) and HCV used are indicated in the upper boxes. The abscissae give the time elapsed between the initiation of the infection and the addition of inhibitors. The experimental points are given in the four panels, and the lines indicate the function that best fits the data. Note that the scale in ordinate differs among panels of the same column. The functions are the following: DAAs with HCV p0 (upper left panel): y=100.00-(5.68·10^−4^e ^(x/0.84)^); DAAs with HCV p200 (upper right panel): y=99.97-(2.21·10^−3^e^(x/0.69)^); mutagens with HCV p0 (bottom left panel): y=99.97-(4.69·10^−3^e^(x/0.60)^); mutagens with HCV p200 (bottom right panel): y=100.00-(6.33·10^−5^e^(x/0.38)^). **(B)** For comparative purposes, the four adjusted functions [color coded as in (A)] are depicted with the same scale in ordinate. Procedures are detailed in Materials and Methods.

The variation of efficacy with time of inhibitor addition following the initiation of infection approximated an exponential function of the form y = y_0_ - Ae^(x/t)^, implying that the decrease of efficacy is more accentuated the longer the infection is allowed to progress before adding the antiviral combinations. The exponential function exhibited a good fit with the experimental points (*R*^2^ ≥ 0.98) (Figure 2A). The A and t terms of the equations reflected a higher inhibition, despite the delayed addition of inhibitors, for the DAAs than for the mutagenic analog combinations (Figure 2B). The delayed administration of DAAs decreased their efficacy more for the high fitness HCV p200 than for HCV p0. No such difference was noted with the mutagenic analog combinations (Figure 2B).

Thus, the addition of DAAs or mutagenic nucleoside analogs to actively replicating HCV infections decreases significantly their antiviral efficacy.

### Extinction of hepatitis C progeny production at 72 h post-initiation of the infection

**Figure 3:**
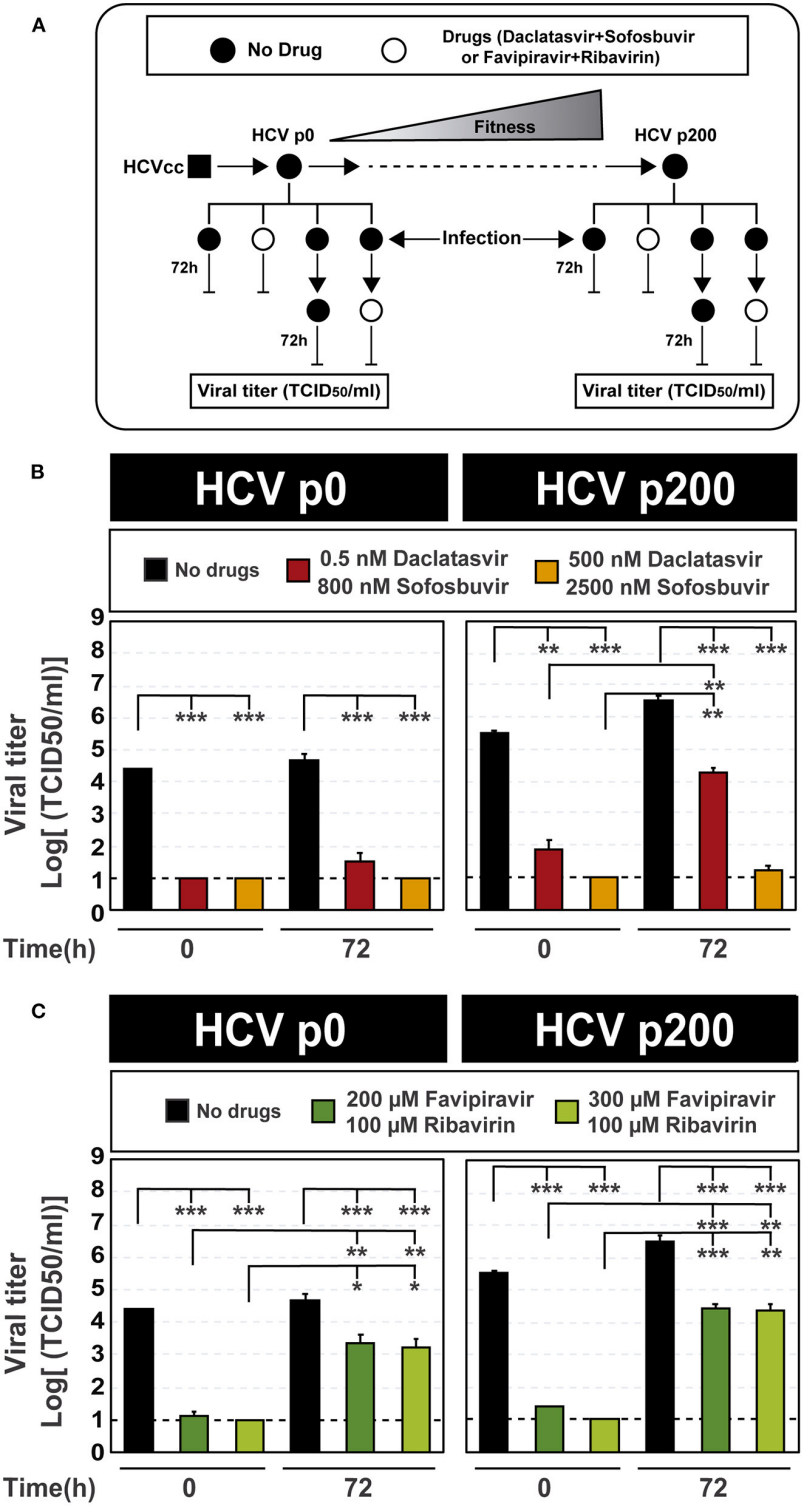
Inhibition of HCV infectious progeny production by combinations of antiviral agents added at the time of infection or 72 h post-infection. **(A)** Scheme of the experiment displaying at the top the evolution of HCV p0 toward its high fitness derivative HCV p200. The time of infection and of addition of the antiviral inhibitors are indicated next to the vertical lines; in all cases, virus titer in the cell culture supernatant was determined 72 h after addition of the inhibitors. **(B)** Virus titer upon the addition of combinations of the DAAs daclatasvir and sofosbuvir (concentrations in the cell culture medium given in the boxes) either at the time of infection or 72 h post-infection (indicated in abscissa); infections were carried out in parallel with HCV p0 and HCV p200. **(C)** Same as **(B)** except that the inhibitors used were combinations of the mutagenic nucleoside analogs favipiravir and ribavirin. For B and C, the statistical significance of the differences between values given in the bars are given as follows: *p < 0.05; **p < 0.01; ***p < 0.001; *t*-test. Experiments were performed in triplicate (replicas A, B, and C in Supplemental Tables S4, S5). Procedures followed for drug preparation, cell infections, and titration of infectivity are detailed in Materials and Methods.

To evaluate conditions that could extinguish high fitness HCV p200, we performed a new experiment by fixing at 72 h the time elapsed between infection initiation and inhibitor addition, and using higher inhibitor concentrations. The maximum concentrations of daclatasvir, sofosbuvir, and favipiravir tested were based on our former results with these inhibitors and HCV (Sheldon et al., 2014; Gallego et al., 2016, 2018), and they were three- to 30-fold lower than their CC_50_ values. Ribavirin was kept at 100 μM because in previous studies this concentration was discriminatory of fitness effects: it produced extinction of HCV p0 after four serial passages, while it failed to extinguish HCV p200 even after ten serial passages (Gallego et al., 2018). Under these treatment conditions (Figure 3A), again, there was a significantly lower inhibition of HCV progeny production when either DAA or lethal mutagen combinations were added at 72 h post-infection, as compared with their addition at the time of infection onset (Figures 3B,C; the numerical values are given in Supplementary Table S4). The decrease of efficacy of the DAAs was more accentuated with HCV p200 than HCV p0, in agreement with the HCV fitness-associated resistance to the DAA inhibitors when used individually (Sheldon et al., 2014; Gallego et al., 2016, 2018). The fitness effect was not noted with the combination of mutagens.

**Figure 4:**
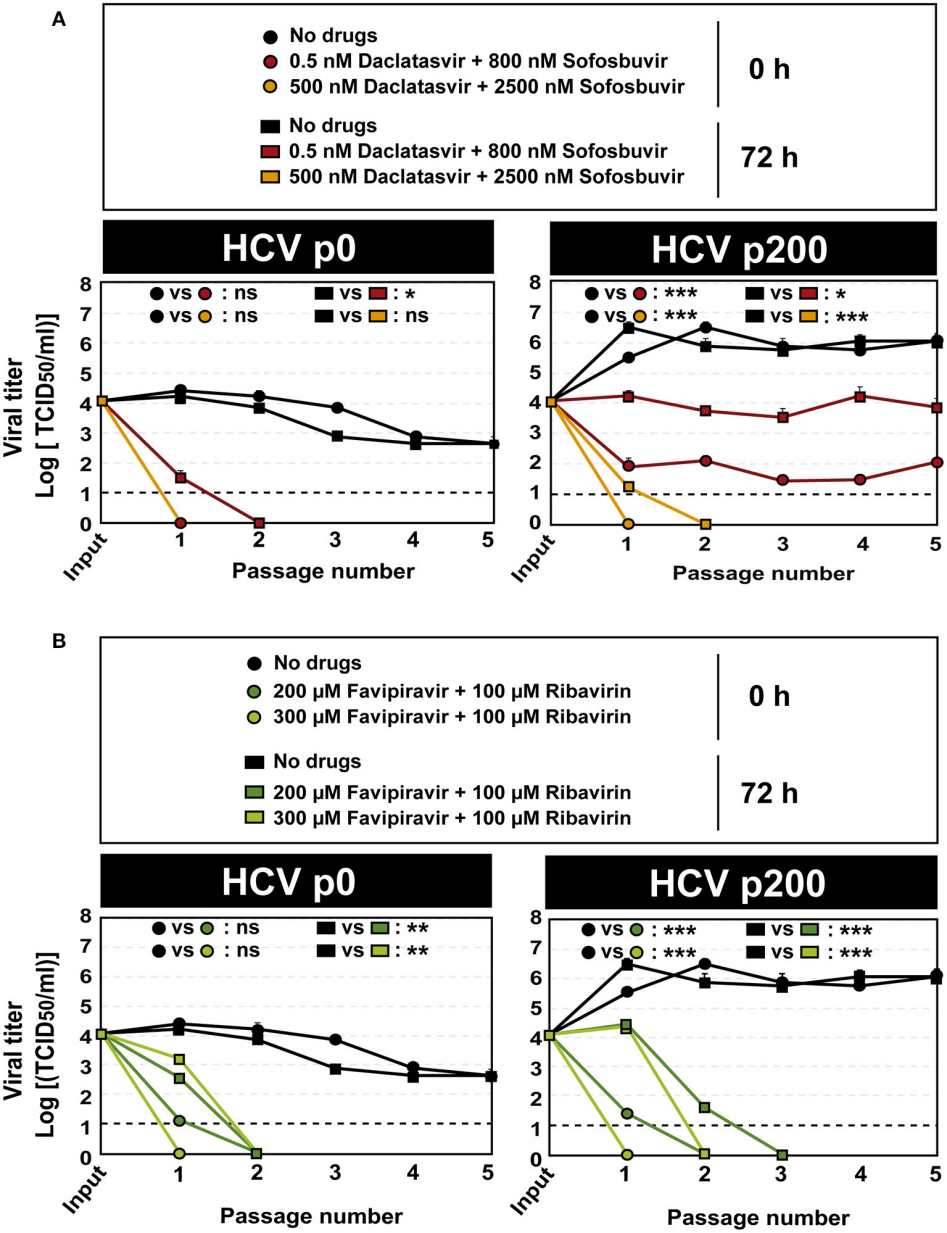
Extinction of HCV populations by serial passages in the presence of antiviral agent combinations. **(A)** The responses of HCV p0 and HCV p200 subjected to five serial infections in the absence or presence of the daclatasvir and sofosbuvir are indicated in the box. **(B)** The responses of HCV p0 and HCV p200 subjected to five serial infections in the absence or presence of the favipiravir and ribavirin are indicated in the box. The initial and the viruses at passage 1 are those described in Figure 3. The time of infection and of addition of the antiviral inhibitors are indicated; in all cases, virus titer in the cell culture supernatant was determined 72 h after addition of the inhibitors. The differences between the viral titers along the passages as given at the top of each graph are as follows: ns: no significant; *p < 0.05; **p < 0.01; ***p < 0.001; ANCOVA test. Viral titer values can be found in Supplemental Table S4. Extracellular RNA values can be found in Supplemental Table S5 and Supplementary Figure S3. Experiments were performed in triplicate (replicas A, B, and C in Supplemental Tables S4, S5). Procedures followed for drug preparation, cell infections, and titration of infectivity are detailed in Materials and Methods.

Since no virus extinction was attained under any of the infection conditions tested in our experiments, we subjected the initial viral populations HCV p0 and HCV p200, as well as the populations obtained at 72h post-infection in the absence and presence of drugs (HCV populations described in Figure 3), to serial infections in the absence and presence of the same drug concentrations. The results indicate that, in all cases, titers below the limit of detection were achieved by passage 3 or earlier, except when the input virus was HCV p200 subjected to 0.5 nM daclatasvir and 800 nM sofosbuvir (Figure 4; the numerical values are given in Supplementary Table S4). For this population, the virus titer remained at around 10^3^-10^5^ TCID_50_/ml for at least five passages (Figure 4A). The inhibitions were confirmed with quantifications of extracellular viral RNA (Supplementary Table S5; Supplementary Figure S3).

To investigate the possible presence of daclatasvir- or sofosbuvir-escape substitutions, the initial HCV p200 population and the populations at passage 5 in the absence and presence of drugs were subjected to Sanger sequencing. The results show several amino acid substitutions whose frequency increased with passage number in the presence of the daclatasvir plus sofosbuvir combination, independently of adding the drugs at 0 or 72 h.p.i. (Supplementary Table S6). Of these substitutions, T24A, F28I, and L31M may account for the lack of effectiveness of the lower DAA concentrations because they have been described as resistance-associated substitutions (RASs) for NS5A inhibitors (Lontok et al., 2015; Sarrazin, 2016; European Association for the Study of the Liver, 2020). The escape effect of these substitutions may be reinforced by the high fitness of HCV p200 (Sheldon et al., 2014; Gallego et al., 2016, 2018; Sabariegos et al., 2022). Some additional substitutions found of HCV p200 passaged in the absence of drugs have been previously described. For example, T245A resulted in a perinuclear-restricted localization of NS5A (Goonawardane et al., 2018), and C465S resulted in an increase of viral titer (Han et al., 2013). No substitutions that coincide with HRSs defined in patients (Soria et al., 2020) have been found in the HCV p200 populations. The same HCV p200 population was effectively extinguished by the higher DAA doses, and all populations were extinguished by the mutagenic analog concentration; no evidence of infectivity or viral RNA was obtained when the populations that yielded a titer below detection level were subjected to the extinction test (Figure 4). Thus, the time post-infection of inhibitor addition is critical to determine inhibitor and lethal mutagenic effectiveness, but continuing treatment may achieve extinction in a modest number of passages.

## Discussion

Models of viral population dynamics predict loss of antiviral efficacy when the inhibitors are added when a viral infection is actively progressing (Nowak and May, 2000; Hadjichrysanthou et al., 2016; Madelain et al., 2018; Goncalves et al., 2020). In this study with HCV in cell culture, we have documented significant reductions of antiviral efficacy correlated with the time of inhibitor addition relative to infection onset. The study has been performed with the well-characterized daclatasvir and sofosbuvir and the lethal mutagens, favipiravir and ribavirin (Perales et al., 2019; Bhattacharjee et al., 2021). The activity of these agents was previously investigated in our laboratory with the clonal population HCV p0 and its high fitness derivative HCV p200 (Ortega-Prieto et al., 2013; Sheldon et al., 2014; de Avila et al., 2016; Gallego et al., 2016, 2018, 2019). This study has confirmed an HCV fitness-mediated antiviral resistance in protocols in which inhibitors were added to an ongoing HCV infection. Due to experimental limitations derived from the number of variables covered, only a reduced range of inhibitor concentrations has been explored, based on CC_50_ and IC_50_ values, as well as on previous analyses with the same inhibitors used individually on inhibition and extinction of HCV p0 (Ortega-Prieto et al., 2013; Sheldon et al., 2014; de Avila et al., 2016; Gallego et al., 2016, 2018). With the inhibitors used, and under the conditions tested, higher viral suppression was achieved with the combination of the DAAs, daclatasvir and sofosbuvir, than with the combination of the mutagens, favipiravir and ribavirin. The relevance of the higher suppression of viral replication attained by these DAAs relative to the nucleoside analog combinations is reinforced by the fact that the concentrations used were on average 12-fold lower than their IC_50_ values for DAAs than for the mutagen combinations (compare Table 1; Figures 1, 2). The higher HCV suppressive effect of the DAAs observed cannot be given the rank of general conclusion on relative efficacy of standard (non-mutagenic) vs. mutagenic inhibitors for HCV. Clarification of this point for HCV will require a comparison of additional DAAs and mutagenic base and nucleoside analogs over a broader range of viral population size and viral fitness.

The exponential functions that relate the percentage of inhibition with the time elapsed between initiation of HCV infection and the addition of inhibitors to the infected cells (Figure 2) suggest that there is a margin of early times after infection in which inhibitor efficacy is minimally affected. Following this initial period, delay in administration has a progressively increasing negative effect on avoiding HCV progeny production. The progressing time-dependent trend of antiviral efficacy loss is in agreement with the competition model proposed to explain the fitness effect of antiviral efficacy (Domingo et al., 2019). According to the competition model, the amount of active forms of the inhibitors per replication complex equivalent may be lower for high fitness HCV. A similar inhibitor limitation effect may occur with an advanced infection as compared with the initial phases of the same infection.

Despite environmental heterogeneity in an *in vivo* setting, the results of our simplified and controlled experimental system are in agreement with observations reported with inhibitor efficacy with several viruses tested in animal models (Mendenhall et al., 2011b; Madelain et al., 2018; Abdelnabi et al., 2021) and with general theoretical predictions (Nowak and May, 2000; Hadjichrysanthou et al., 2016).

The consequences of delayed treatment implementation bear indirectly on the issue of at which point in the development of HCV-related hepatic disease (i.e. liver fibrosis stage) should an antiviral treatment be initiated (Chahal et al., 2016; El Sayed et al., 2017; Ruggeri et al., 2018). Long-term HCV infection does not necessarily mean a steady increase of viral load due to the many host and viral factors involved, as well as viral turnover and clearance (Dahari et al., 2005). However, it is expected that prolonged replication in the same liver may favor fitness increase of the resident HCV. This emphasizes the success of current DAA-based treatments of chronic HCV infection, which achieve sustained virological responses of around 95% (Janjua et al., 2021). The process required for the approval of new antiviral agents (even if they are a modified form of a previously licensed agent or one investigated from drug repositioning) is generally lengthy and costly. Therefore, we suggest that the preclinical screening of antiviral agents in cell culture would benefit from using high fitness viral populations and administration of the agents once the infection is underway. High fitness viral populations can presently be derived in a straightforward manner provided the host cell or organism—in which the antiviral tests will be conducted—is available to support serial virus passages (Domingo and Holland, 1997). A multiple passaged virus acquires significant fitness increases relative to the parental population when fitness is measured in the same environment (Domingo et al., 2022). The influence of high HCV fitness on the decrease of inhibitory activity was noted with both combinations, as expected from previous results on the higher effectiveness of extinction of low fitness HCV than high fitness HCV by the individual antiviral agents (Sheldon et al., 2014; Gallego et al., 2016, 2018). With the combinations used in this study, the efficacy decrease associated with high HCV fitness was clearly noted with the DAAs and far less with the lethal mutagens (Figures 1, 3). In the plots that represented the decrease of inhibitory activity as a function of the time post-infection of inhibitors administration, an attenuating effect of HCV fitness on the loss of inhibition was noted with the DAA combination and not with the mutagen combination (Figure 2). Additional data with other inhibitors and lethal mutagens are needed to suggest if this can be an advantage of lethal mutagens vs. non-mutagenic inhibitors for HCV and other RNA viruses. Protocols using high fitness virus and delayed administration of inhibitors (either individually or in combination) provide a better filter to avoid expensive biological assays prior to clinical tests with humans.

## Data availability statement

The original contributions presented in the study are included in the article/Supplementary material, further inquiries can be directed to the corresponding authors.

## Ethics statement

This study used human cell lines obtained from Dr. Charles Rice's laboratory. The institutional Bioethics Committee from Consejo Superior de Investigaciones Científicas (CSIC), in accord with Spanish regulations did not require the study to be reviewed or approved, because only established cell lines and no human samples were involved.

## Author contributions

ED and CP conceived, designed, and supervised the project. CG-C, LV-S, PS, MS, IG, AÁ, and AD-P performed the experiments and analyzed the results. BM-G performed the statistical analyses. ED, CP, and CG-C wrote the manuscript. All authors contributed to the article and approved the submitted version.

## Funding

This work was supported by Instituto de Salud Carlos III, Spanish Ministry of Science and Innovation (COVID-19 Research Call COV20/00181) and cofinanced by European Development Regional Fund A way to achieve Europe and grants CSIC-COV19-014 from Consejo Superior de Investigaciones Científicas (CSIC), project 525/C/2021 from Fundació La Marató de TV3, PID2020-113888RB-I00 from Ministerio de Ciencia e Innovación, BFU2017-91384-EXP from Ministerio de Ciencia, Innovación y Universidades (MCIU), PI18/00210 and PI21/00139 from Instituto de Salud Carlos III, and S2018/BAA-4370 (PLATESA2 from Comunidad de Madrid/FEDER). This research work was also funded by the European Commission NextGenerationEU (Regulation EU 2020/2094), through the CSIC's Global Health Platform (PTI Salud Global). CP was supported by the Miguel Servet Program of the Instituto de Salud Carlos III (CP14/00121 and CPII19/00001), cofinanced by the European Regional Development Fund (ERDF). Centro de Investigación en Red de Enfermedades Hepáticas y Digestivas (CIBERehd) is funded by Instituto de Salud Carlos III. Institutional grants from the Fundación Ramón Areces and Banco Santander to the CBMSO are also acknowledged. The team at CBMSO belongs to the Global Virus Network (GVN). CG-C was supported by predoctoral contract PRE2018-083422 from MCIU. PS was supported by postdoctoral contract Margarita Salas CA1/RSUE/2021 from MCIU. BM-G was supported by predoctoral contract PFIS FI19/00119 from ISCIII, cofinanced by Fondo Social Europeo (FSE).

## Conflict of interest

The authors declare that the research was conducted in the absence of any commercial or financial relationships that could be construed as a potential conflict of interest.

## Publisher's note

All claims expressed in this article are solely those of the authors and do not necessarily represent those of their affiliated organizations, or those of the publisher, the editors and the reviewers. Any product that may be evaluated in this article, or claim that may be made by its manufacturer, is not guaranteed or endorsed by the publisher.
