# Supplementary material for: Efficacy decrease of antiviral agents when administered to ongoing hepatitis C virus infections in cell culture
Source: Front Microbiol. 2022 Aug 3;13:960676. doi: 10.3389/fmicb.2022.960676 (PMC9382109; doi:10.3389/fmicb.2022.960676)
Supplement: Supplementary file 1 [file Data_Sheet_1.PDF]

Supplemental material:

Efficacy decrease of antiviral agents when administered to ongoing hepatitis C virus infections

Carlos García-Crespo[1,2], Lucía Vázquez-Sirvent[1,3], Pilar Somovilla[1,4], María Eugenia Soria[1,2,3], Isabel Gallego[1,2], Ana Isabel de Ávila[1,2], Brenda Martínez-González[3,5], Antoni Durán-Pastor[1], Esteban Domingo[1,2]* and Celia Perales[1,2,3,5]*

[1]*Centro de Biología Molecular "Severo Ochoa" (CSIC-UAM), Consejo Superior de Investigaciones Científicas (CSIC), Campus de Cantoblanco, 28049, Madrid, Spain,* [2]*Centro de Investigación Biomédica en Red de Enfermedades Hepáticas y Digestivas (CIBERehd) del Instituto de Salud Carlos III, 28029, Madrid, Spain,* [3]*Department of Clinical Microbiology, IIS-Fundación Jiménez Díaz, UAM. Av. Reyes Católicos 2, 28040 Madrid, Spain,* [4]*Departamento de Biología Molecular, Universidad Autónoma de Madrid, Campus de Cantoblanco, 28049, Madrid, Spain,* [5]*Department of Molecular and Cell Biology, Centro Nacional de Biotecnología (CNB-CSIC), Consejo Superior de Investigaciones Científicas (CSIC), Campus de Cantoblanco, 28049 Madrid, Spain*

Running title: Efficacy of anti-HCV agents

*Corresponding authors:

E-mails addresses: Esteban Domingo (edomingo@cbm.csic.es) and Celia Perales (celia.perales@cnb.csic.es)

TABLE S1. Oligonucleotides used to amplify and sequence the HCV genomes.

Primer Name	Sequence (5'-3')	Position[a]
**HCV-5UTR-F2**	TGAGGAACTACTGTCTTCACGCAGAAAG	47-74
**HCV-5UTR-R2**	TGCTCATGGTGCACGGTCTACGAG	324-347
**Jc1-NS5A F1**	ACTCAGAAGACTCCACAATTGG	6220-6241
**Jc1-NS5A F2**	ACTACCTTCTCCAGAGTTTTTC	6697-6718
**Jc1-NS5A R1**	TGGGTGCAAACCTATGGATCTG	6737-6758
**Jc1-NS5A F3**	TTTCCACGGGCCTTACCGGCTTG	7181-7203
**Jc1-NS5A T5F**	TGGGCACGGCCTGACTACAACC	7202-7223
**Jc1-NS5A R2**	ACGATTCCACGAGCGGCGGGTTG	7219-7241
**Jc1-NS5B F1**	TGGTCTACTTGCTCCGAGGAGG	7625-7646
**Jc1-NS5A R3**	ACTCAAAGGGTTGATTGGCAAC	7726-7747
**Jc1-NS5B F2**	TAAGAAACCAGCTCGCCTCATC	8125-8146
**Jc1-NS5B R1**	TCTCGCAGACCCGGACGCCGAG	8159-8180
**Jc1-NS5B F3**	TGCGCCCACAATGCTGGTATGC	8593-8614
**Jc1-NS5B R5**	ACCTGGTCATGGCCTCCGTG	8683-8702
**Jc1-NS5B F4**	AACCTCAACTTTGAGATGTATG	8990-9011
**Jc1-NS5B-R3**	TCTCAATTATGGCTGGAAGGTCC	9037-9059
**Jc1-NS5B R4**	AGTTAGCTATGGAGTGTACCTAG	9454-9476

[a] Residue numbering is according to JFH-1, GenBank accession number # AB047639.

TABLE S2. Viral titer ($TCID_{50}$/ml) values represented in Figures 1 and 2 of the main text.

Virus[a]	Treatment[b]	Replica[c]	Time of infection before adding the antivirals			
			**0h**	**24h**	**48h**	**72h**
HCV p0	No drugs	A	1.31×10^4	1.95×10^4	2.68×10^4	2.15×10^4
		B	2.25×10^4	4.64×10^4	5.13×10^4	3.73×10^4
		C	3.98×10^4	3.73×10^4	1.58×10^5	7.63×10^4
HCV p0	0.5 nM Daclastasvir + 800 nM Sofosbuvir	A	<10	<10	1.95 x 10	<10
		B	<10	<10	<10	5.8 x 10
		C	<10	<10	<10	2.99 x 10
HCV p0	200 µM Favipiravir + 100 µM Ribavirin	A	1.95 x 10	1.95 x 10	3.98×10^2	3.73×10^2
		B	<10	1.95 x 10	5.80 x 10	1.95×10^3
		C	<10	1.95 x 10	1.00×10^3	2.68×10^3
HCV p200	No drugs	A	1.31×10^5	2.28×10^6	1.58×10^6	1.00×10^6
		B	2.68×10^5	4.64×10^6	4.64×10^6	5.13×10^6
		C	3.89×10^5	4.64×10^6	2.68×10^6	3.16×10^6
HCV p200	0.5 nM Daclastasvir + 800 nM Sofosbuvir	A	2.43 x 10	1.26×10^3	5.13×10^3	1.26×10^4
		B	1.58×10^2	1.95×10^3	4.64×10^3	2.68×10^4
		C	5.80 x 10	1.58×10^3	5.62×10^3	1.31×10^4
HCV p200	200 µM Favipiravir + 100 µM Ribavirin	A	1.95 x 10	1.95×10^2	3.73×10^3	4.64×10^4
		B	1.95 x 10	7.94×10^2	6.40×10^3	3.73×10^4
		C	<10	6.31×10^2	4.39×10^3	6.31×10^4

[a]Virus used in the experiment.

[b]Concentration of antivirals used.

[c]Biological triplicate.

TABLE S3. Extracellular RNA (molecules/ml) values represented in **Figure S2**.

Virus[a]	Treatment[b]	Replica[c]	Time of infection before adding the antivirals			
			0h	24h	48h	72h
HCV p0	No drugs	A	4.87×10^{7}	3.07×10^{8}	7.36×10^{8}	8.71×10^{8}
		B	5.36×10^{7}	2.90×10^{8}	8.51×10^{8}	1.98×10^{9}
		C	5.92×10^{7}	4.66×10^{8}	8.07×10^{8}	1.56×10^{9}
HCV p0	0.5 nM Daclatasvir + 800 nM Sofosbuvir	A	1.91×10^{5}	6.27×10^{5}	4.24×10^{6}	2.00×10^{7}
		B	2.06×10^{5}	5.68×10^{5}	4.84×10^{6}	2.06×10^{7}
		C	2.26×10^{5}	4.69×10^{5}	3.71×10^{6}	2.21×10^{7}
HCV p0	200 µM Favipiravir + 100 µM Ribavirin	A	4.76×10^{5}	3.59×10^{6}	1.69×10^{7}	1.26×10^{8}
		B	4.53×10^{5}	2.34×10^{6}	1.94×10^{7}	1.96×10^{8}
		C	3.30×10^{5}	3.67×10^{6}	2.46×10^{7}	1.70×10^{8}
HCV p200	No drugs	A	4.06×10^{8}	3.13×10^{9}	2.50×10^{9}	1.75×10^{8}
		B	4.68×10^{8}	2.62×10^{9}	9.51×10^{8}	3.45×10^{9}
		C	5.61×10^{8}	2.23×10^{9}	8.08×10^{9}	5.21×10^{9}
HCV p200	0.5 nM Daclatasvir + 800 nM Sofosbuvir	A	5.55×10^{5}	3.45×10^{6}	3.00×10^{7}	2.40×10^{8}
		B	8.19×10^{5}	3.59×10^{6}	4.19×10^{6}	1.63×10^{8}
		C	6.15×10^{5}	1.20×10^{6}	4.25×10^{7}	8.37×10^{8}
HCV p200	200 µM Favipiravir + 100 µM Ribavirin	A	6.01×10^{5}	6.77×10^{5}	6.78×10^{7}	2.22×10^{8}
		B	7.06×10^{5}	7.58×10^{6}	5.84×10^{6}	4.32×10^{7}
		C	1.27×10^{6}	3.73×10^{6}	3.78×10^{7}	6.53×10^{8}

[a]Virus used in the experiment.

[b]Concentration of antivirals used.

[c]Biological triplicate.

TABLE S4. Viral titer ($TCID_{50}$/mL) values represented in **Figures 3 and 4**.

Virus[a]	Treatment[b]	Time[c]	Replica[d]	Viral titer ($TCID_{50}$/mL)				
				**Passage 1**	**Passage 2**	**Passage 3**	**Passage 4**	**Passage 5**
HCV p0	No drugs	0h	A	2.68×10^4	2.15×10^4	1.00×10^4	6.31×10^2	4.15×10^2
			B	2.57×10^4	3.73×10^4	6.51×10^3	1.00×10^3	5.01×10^2
			C	2.51×10^4	7.63×10^4	5.62×10^3	6.31×10^2	3.67×10^2
HCV p0	0.5 nM DCV + 800 nM SOF	0h	A	<10	<10	<10	<10	<10
			B	<10	<10	<10	<10	<10
			C	<10	<10	<10	<10	<10
HCV p0	500 nM DCV + 2500 nM SOF	0h	A	<10	<10	<10	<10	<10
			B	<10	<10	<10	<10	<10
			C	<10	<10	<10	<10	<10
HCV p0	200 µM FVP + 100 µM Rib	0h	A	<10	<10	<10	<10	<10
			B	<10	<10	<10	<10	<10
			C	1.95 x 10	<10	<10	<10	<10
HCV p0	300 µM FVP + 100 µM Rib	0h	A	<10	<10	<10	<10	<10
			B	<10	<10	<10	<10	<10
			C	<10	<10	<10	<10	<10
HCV p0	No drugs	72h	A	2.15×10^4	1.00×10^4	6.31×10^2	4.15×10^2	7.61×10^2
			B	3.73×10^4	6.51×10^3	1.00×10^3	5.01×10^2	2.32×10^2
			C	7.63×10^4	5.62×10^3	6.31×10^2	3.67×10^2	2.98×10^2
HCV p0	0.5 nM DCV + 800 nM SOF	72h	A	<10	<10	<10	<10	<10
			B	5.80 x 10	<10	<10	<10	<10
			C	2.99 x 10	<10	<10	<10	<10
HCV p0	500 nM DCV + 2500 nM SOF	72h	A	<10	<10	<10	<10	<10
			B	<10	<10	<10	<10	<10
			C	<10	<10	<10	<10	<10
HCV p0	200 µM FVP + 100 µM Rib	72h	A	6.40×10^2	<10	<10	<10	<10
			B	2.68×10^3	<10	<10	<10	<10
			C	3.73×10^3	<10	<10	<10	<10
HCV p0	300 µM FVP + 100 µM Rib	72h	A	1.00×10^3	<10	<10	<10	<10
			B	7.94×10^2	<10	<10	<10	<10
			C	3.06×10^3	<10	<10	<10	<10
HCV p200	No drugs	0h	A	3.73×10^5	4.64×10^6	3.73×10^5	4.64×10^5	1.70×10^6
			B	3.73×10^5	3.73×10^6	3.73×10^5	3.16×10^5	1.58×10^6
			C	2.63×10^5	1.58×10^6	1.58×10^6	1.00×10^6	3.73×10^5
HCV p200	0.5 nM DCV + 800 nM SOF	0h	A	2.43 x 10	5.80 x 10	2.43 x 10	2.43 x 10	1.58×10^2
			B	1.58 x 10	1.58×10^2	2.99 x 10	4.44 x 10	1.58×10^2
			C	5.80 x 10	1.58×10^2	2.43 x 10	2.43 x 10	1.95 x 10
HCV p200	500 nM DCV + 2500 nM SOF	0h	A	<10	<10	<10	<10	<10
			B	<10	<10	<10	<10	<10
			C	<10	<10	<10	<10	<10
HCV p200	200 µM FVP + 100 µM Rib	0h	A	2.43 x 10	<10	<10	<10	<10
			B	2.43 x 10	<10	<10	<10	<10
			C	2.43 x 10	<10	<10	<10	<10

HCV p200	300 µM FVP + 100 µM Rib	0h	A	<10	<10	<10	<10	<10
			B	<10	<10	<10	<10	<10
			C	<10	<10	<10	<10	<10
HCV p200	No drugs	72h	A	4.64×10^6	3.73×10^5	4.64×10^5	1.70×10^6	1.00×10^6
			B	3.73×10^6	3.73×10^5	3.16×10^5	1.58×10^6	3.16×10^5
			C	1.58×10^6	1.58×10^6	1.00×10^6	3.73×10^5	2.13×10^6
HCV p200	0.5 nM DCV + 800 nM SOF	72h	A	1.26×10^4	5.13×10^3	6.31×10^3	3.73×10^4	1.58×10^4
			B	2.68×10^4	3.16×10^3	4.64×10^2	1.93×10^3	4.64×10^2
			C	1.31×10^4	7.63×10^3	3.73×10^3	1.31×10^4	5.62×10^3
HCV p200	500 nM DCV + 2500 nM SOF	72h	A	1.95 x 10	<10	<10	<10	<10
			B	<10	<10	<10	<10	<10
			C	<10	<10	<10	<10	<10
HCV p200	200 µM FVP + 100 µM Rib	72h	A	1.95×10^4	3.16 x 10	<10	<10	<10
			B	3.98×10^4	4.44 x 10	<10	<10	<10
			C	2.15×10^4	3.98 x 10	<10	<10	<10
HCV p200	300 µM FVP + 100 µM Rib	72h	A	3.73×10^4	<10	<10	<10	<10
			B	2.57×10^4	<10	<10	<10	<10
			C	1.00×10^4	<10	<10	<10	<10

[a]Virus used in the experiment.

[b]Concentration of antivirals used. DCV: daclatasvir; SOF: sofosbuvir; FVP: favipiravir; Rib: ribavirin.

[c]Time at which antivirals are added.

[d]Biological triplicate.

TABLE S5. Extracellular RNA (molecules/ml) values represented in **Figure S3**.

Virus[a]	Treatment[b]	Time[c]	Replica[d]	Extracellular RNA (molecules/ml)				
				Passage 1	Passage 2	Passage 3	Passage 4	Passage 5
HCV p0	No drugs	0h	A	9.78×10^7	4.09×10^8	4.65×10^7	1.10×10^7	5.05×10^5
			B	9.89×10^7	3.44×10^8	4.89×10^7	6.45×10^6	7.27×10^5
			C	1.01×10^8	2.24×10^8	3.57×10^7	7.59×10^6	7.79×10^5
HCV p0	0.5 nM DCV + 800 nM SOF	0h	A	1.91×10^5				
			B	2.06×10^5	Extinction	Extinction	Extinction	Extinction
			C	2.26×10^5				
HCV p0	500 nM DCV + 2500 nM SOF	0h	A	3.66×10^5				
			B	1.89×10^5	Extinction	Extinction	Extinction	Extinction
			C	1.89×10^5				
HCV p0	200 µM FVP + 100 µM Rib	0h	A	1.77×10^6	7.75×10^4			
			B	1.13×10^6	4.94×10^5	Extinction	Extinction	Extinction
			C	1.08×10^6	5.36×10^5			
HCV p0	300 µM FVP + 100 µM Rib	0h	A	5.37×10^5				
			B	6.52×10^5	Extinction	Extinction	Extinction	Extinction
			C	7.23×10^5				
HCV p0	No drugs	72h	A	4.09×10^8	4.65×10^7	1.10×10^7	5.05×10^5	2.49×10^5
			B	3.44×10^8	4.89×10^7	6.45×10^6	7.27×10^5	5.60×10^5
			C	2.24×10^8	3.57×10^7	7.59×10^6	7.79×10^5	5.27×10^5
HCV p0	0.5 nM DCV + 800 nM SOF	72h	A	2.00×10^7	Extinction			
			B	2.06×10^7	1.26×10^5	Extinction	Extinction	Extinction
			C	2.21×10^7	8.42×10^5			
HCV p0	500 nM DCV + 2500 nM SOF	72h	A	8.58×10^6				
			B	9.03×10^6	Extinction	Extinction	Extinction	Extinction
			C	3.53×10^6				
HCV p0	200 µM FVP + 100 µM Rib	72h	A	1.54×10^8	1.00×10^5			
			B	1.92×10^8	1.08×10^5	Extinction	Extinction	Extinction
			C	1.37×10^8	1.09×10^5			
HCV p0	300 µM FVP + 100 µM Rib	72h	A	1.59×10^7	1.95×10^5			
			B	2.50×10^7	5.95×10^5	Extinction	Extinction	Extinction
			C	2.18×10^7	6.11×10^5			
HCV p200	No drugs	0h	A	5.17×10^8	1.13×10^{10}	2.19×10^9	4.03×10^9	8.84×10^8
			B	5.39×10^8	3.11×10^9	3.43×10^9	2.55×10^9	8.30×10^8
			C	5.81×10^8	1.01×10^9	5.88×10^9	2.82×10^9	7.17×10^8
HCV p200	0.5 nM DCV + 800 nM SOF	0h	A	5.55×10^5	1.91×10^5	2.17×10^5	2.21×10^5	2.17×10^5
			B	8.19×10^5	3.71×10^5	3.09×10^5	2.99×10^5	2.74×10^5
			C	6.15×10^5	1.31×10^5	2.23×10^5	1.98×10^5	1.81×10^5
HCV p200	500 nM DCV + 2500 nM SOF	0h	A	2.51×10^5				
			B	2.76×10^5	Extinction	Extinction	Extinction	Extinction
			C	1.50×10^5				
HCV p200	200 µM FVP + 100 µM Rib	0h	A	8.76×10^5	1.23×10^5			
			B	9.93×10^5	4.81×10^5	Extinction	Extinction	Extinction
			C	9.72×10^5	6.12×10^5			

HCV p200	300 µM FVP + 100 µM Rib	0h	A	3.46×10^{5}	Extinction	Extinction	Extinction	Extinction
			B	5.47×10^{5}				
			C	5.93×10^{5}				
HCV p200	No drugs	72h	A	1.13×10^{10}	2.19×10^{9}	4.03×10^{9}	8.84×10^{8}	3.86×10^{8}
			B	3.11×10^{9}	3.43×10^{9}	2.55×10^{9}	8.30×10^{8}	2.61×10^{8}
			C	1.01×10^{9}	5.88×10^{9}	2.82×10^{9}	7.17×10^{8}	3.14×10^{8}
HCV p200	0.5 nM DCV + 800 nM SOF	72h	A	2.40×10^{8}	8.37×10^{6}	6.12×10^{6}	1.10×10^{7}	1.64×10^{7}
			B	1.63×10^{8}	5.36×10^{6}	1.41×10^{6}	1.12×10^{6}	7.04×10^{5}
			C	8.37×10^{8}	1.23×10^{7}	7.72×10^{6}	3.04×10^{7}	2.52×10^{7}
HCV p200	500 nM DCV + 2500 nM SOF	72h	A	1.05×10^{8}	5.79×10^{5}	Extinction	Extinction	Extinction
			B	6.09×10^{7}	3.43×10^{5}			
			C	1.58×10^{8}	3.53×10^{5}			
HCV p200	200 µM FVP + 100 µM Rib	72h	A	1.30×10^{9}	3.55×10^{6}	7.15×10^{4}	Extinction	Extinction
			B	1.29×10^{8}	1.83×10^{6}	3.59×10^{5}		
			C	3.79×10^{8}	1.94×10^{6}	6.59×10^{4}		
HCV p200	300 µM FVP + 100 µM Rib	72h	A	4.79×10^{8}	3.32×10^{5}	Extinction	Extinction	Extinction
			B	3.17×10^{8}	7.63×10^{5}			
			C	3.17×10^{8}	8.42×10^{5}			

[a]Virus used in the experiment.

[b]Concentration of antivirals used. DCV: Daclatasvir; SOF: Sofosbuvir; FVP: Favipiravir; Rib: Ribavirin.

[c]Time at which antivirals are added.

[d]Biological triplicate.

TABLE S6. Mutations and corresponding amino acid substitutions in the NS5A-NS5B coding region analyzed by Sanger sequencing.

Viral population[a]										Amino Acid Substitution[e]	Protein
Initial HCV p200[b]	HCV p200 p5[c]			HCV p200 p5 (0.5 nM Daclatasvir + 800 nM Sofosbuvir added at 0h[d])			HCV p200 p5 (0.5 nM Daclatasvir + 800 nM Sofosbuvir added at 72h[d])				
	Replica A	Replica B	Replica C	Replica A	Replica B	Replica C	Replica A	Replica B	Replica C		
A6338G	A6338G	A6338G	A6338G							**T24A**	**NS5A**
				U6350A	U6350A	U6350A	U6350A	U6350A	U6350A	**F28I**	
				C6359A	C6359A	C6359A	C6359A	C6359A	C6359A	**L31M**	
			C6376U	C6376U	C6376U	C6376U	C6376U	C6376U	C6376U	**-**	
C6412A	C6412A	C6412A	C6412A							**-**	
A6491G	A6491G	A6491G	A6491G							**T75A**	
U6532C	U6532C	U6532C	U6532C							**-**	
A6636G	A6636G	A6636G	A6636G							**Q123R**	
A6658G	A6658G	A6658G	A6658G							**-**	
G6748A	G6748A	G6748A	G6748A							**-**	
				U6883C	U6883C	U6883C	U6883C		U6883C	**-**	
				G6898C	G6898C	G6898C	G6898C		G6898C	**-**	
	C6968A		C6968A	C6968A	C6968A	C6968A	C6968A	C6968A	C6968A	**L234I**	
C6999U	C6999U	C6999U	C6999U							**T244I**	
A7001G	A7001G	A7001G	A7001G							**T245A**	
				C7009G	C7009G	C7009G	C7009G	C7009G	C7009G	**S247R**	

				U7032C	U7032C	U7032C	U7032C		U7032C	**V255A**	
				G7081U	G7081U	G7081U	G7081U	G7081U	G7081U	**E271D**	
				C7083U	C7083U	C7083U	C7083U	C7083U	C7083U	**S272P**	
				A7101G	A7101G	A7101G	A7101G	A7101G	A7101G	**D278G**	
U7107C		U7107C	U7107C							**L280P**	
U7160C	U7160C	U7160C	U7160C	U7160C	U7160C	U7160C	U7160C	U7160C	U7160C	**C298R**	
A7175G		A7175G								**S303G**	
		U7181C	U7181C							**F305L**	
					A7186G					**-**	
A7218G	A7218G	A7218G	A7218G							**Y317C**	
				U7238A	U7238A	U7238A	U7238A	U7238A	U7238A	**S324T**	
A7302G	A7302G	A7302G	A7302G							**K345R**	
				G7312A	G7312A	G7312A	G7312A		G7312A	**-**	**NS5A**
				A7321G	A7321G	A7321G	A7321G		A7321G	**-**	
A7325G	A7325G	A7325G	A7325G							**R353G**	
G7345C	G7345C	G7345C	G7345C							**-**	
A7360G	A7360G	A7360G	A7360G	A7360G	A7360G	A7360G	A7360G	A7360G	A7360G	**I364M**	
				G7425A	G7425A	G7425A	G7425A	G7425A	G7425A	**G386D**	
				C7444U	C7444U	C7444U	C7444U	C7444U	C7444U	**-**	
				A7452G	A7452G	A7452G	A7452G	A7452G	A7452G	**E395G**	
A7453G	A7453G	A7453G	A7453G	A7453G	A7453G	A7453G	A7453G	A7453G	A7453G	**-**	
				U7454C	U7454C	U7454C	U7454C		U7454C	**S396P**	
A7494G	A7494G	A7494G	A7494G							**E409G**	
				A7498G	A7498G	A7498G	A7498G	A7498G	A7498G	**-**	
G7499A	G7499A	G7499A	G7499A							**G411S**	

							G7601C	G7601C		**A445P**	
U7610C	U7610C	U7610C								**S448P**	
				G7618A	G7618A	G7618A	G7618A	G7618A	G7618A	-	
				A7652G	A7652G	A7652G	A7652G	A7652G	A7652G	**T462A**	**NS5A**
				A7655U	A7655U	A7655U	A7655U	A7655U	A7655U	**T463S**	
U7661A	U7661A		U7661A							**C465S**	
				A7789G	A7789G	A7789G	A7789G	A7789G	A7789G	-	
				U7842C	U7842C	U7842C	U7842C	U7842C	U7842C	**V59A**	
U7942C	U7942C	U7942C	U7942C							-	
				C8071U	C8071U	C8071U	C8071U	C8071U	C8071U	-	
C8132U	C8132U	C8132U	C8132U							**P156S**	
						U8137C				-	
G8209U	G8209U	G8209U	G8209U							**K181N**	
U8353C	U8353C	U8353C	U8353C							-	
			U8400C							**L245P**	
C8422U	C8422U	C8422U	C8422U							-	**NS5B**
				A8427G	A8427G	A8427G	A8247G	A8427G	A8427G	**H254R**	
				U8446G	U8446G		U8446G	U8446G	U8446G	-	
				A8475G	A8475G	A8475G	A8475G	A8475G	A8475G	**K270R**	
				C8575U	C8575U	C8575U	C8575U	C8575U	C8575U	-	
U8722A		U8722A	U8722A							**D352E**	
A8752G	A8752G	A8752G	A8752G							-	
A8758G	A8758G	A8758G	A8758G							-	
C8842U	C8842U	C8842U	C8842U	C8842U	C8842U	C8842U	C8842U	C8842U	C8842U	-	
U8884C	U8884C	U8884C								-	

				G8893A	G8893A	G8893A	G8893A	G8893A	G8893A	-	
U8929C	U8929C	U8929C	U8929C							-	**NS5B**
U8962A	U8962A	U8962A	U8962A	U8962A	U8962A	U8962A	U8962A	U8962A	U8962A	-	
				A9235G	A9235G	A9235G	A9235G		A9235G	-	

[a]Viral populations used in the experiment.

[b]Initial virus used for infections in the experiments.

[c]Virus HCV p200 after 5 passages in human hepatoma cells.

[d]Virus HCV p200 after 5 passages in human hepatoma cells in the presence of antivirals.

[e]Amino acid residues (single letter code) are numbered from N- to the C-terminus of NS5A-NS5B.

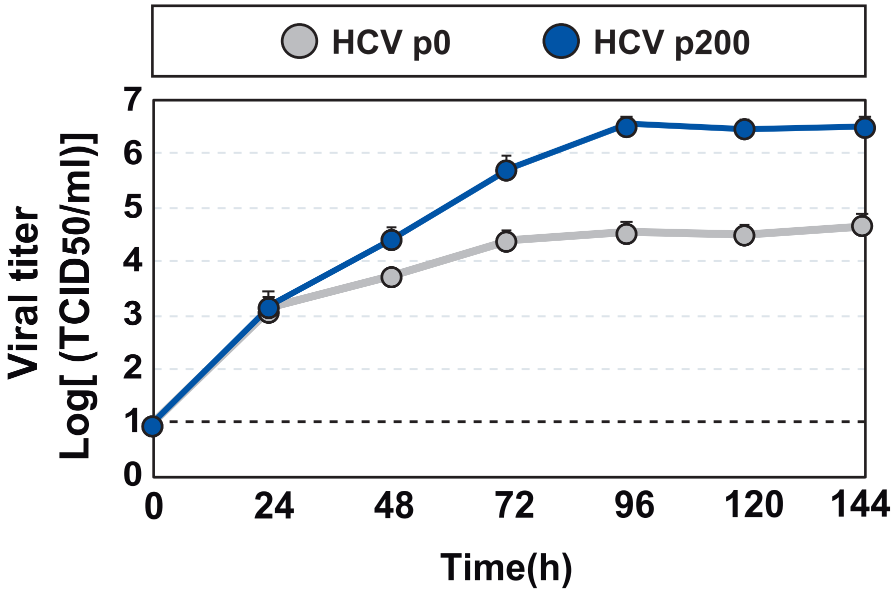

FIGURE S1. Kinetics of infectious progeny production upon infection of Huh-7.5 reporter cells by either HCV p0 or HCV p200 at a multiplicity of infection (MOI) of 0.03 $TCID_{50}$/cell.

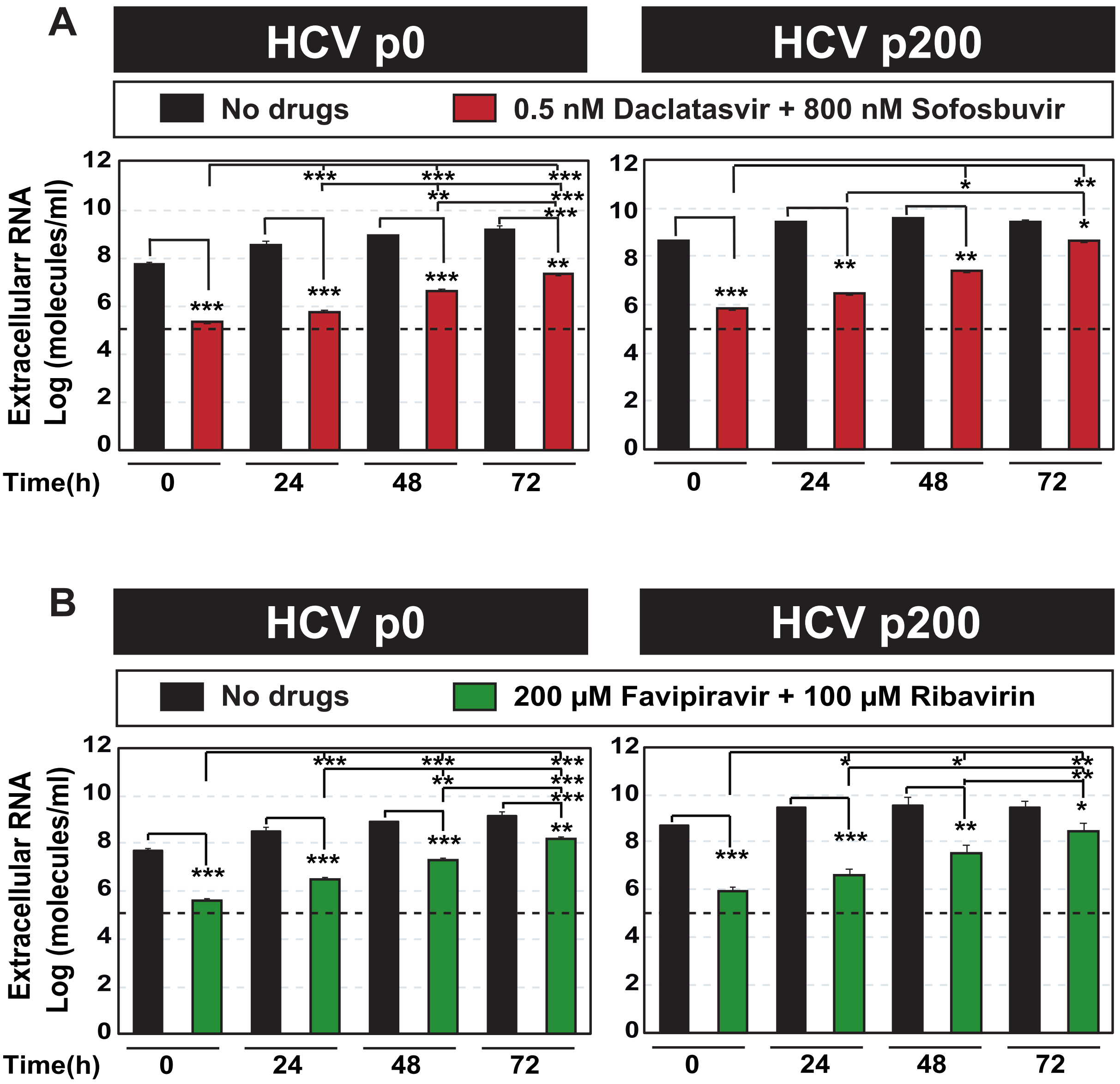

FIGURE S2. Effect of the time of addition of inhibitor combinations after initiation of HCV infection on extracellular RNA. The time of infection and of addition of the inhibitors are indicated; in all cases, extracellular RNA was extracted from cell culture supernatants and RNA was quantified at 72h following the last addition of inhibitors. **(A)** Extracellular RNA upon addition of combinations of the DAAs daclatasvir and sofosbuvir. The virus used for infection is indicated in the upper filled boxes, and the inhibitor concentrations in the cell culture medium are given in the empty box. The abscissa shows the time post-infection of addition of the drug combination. **(B)** Same as (A) except that the inhibitors used were combinations of the mutagenic nucleoside analogues favipiravir and ribavirin. For A, B, the statistical significance of the differences between values given in the bars are given as follows: * *P*<0.05; ***P*<0.01; ****P*<0.001; t-test). Extracellular RNA values can be found in **Supplemental Table S3**.

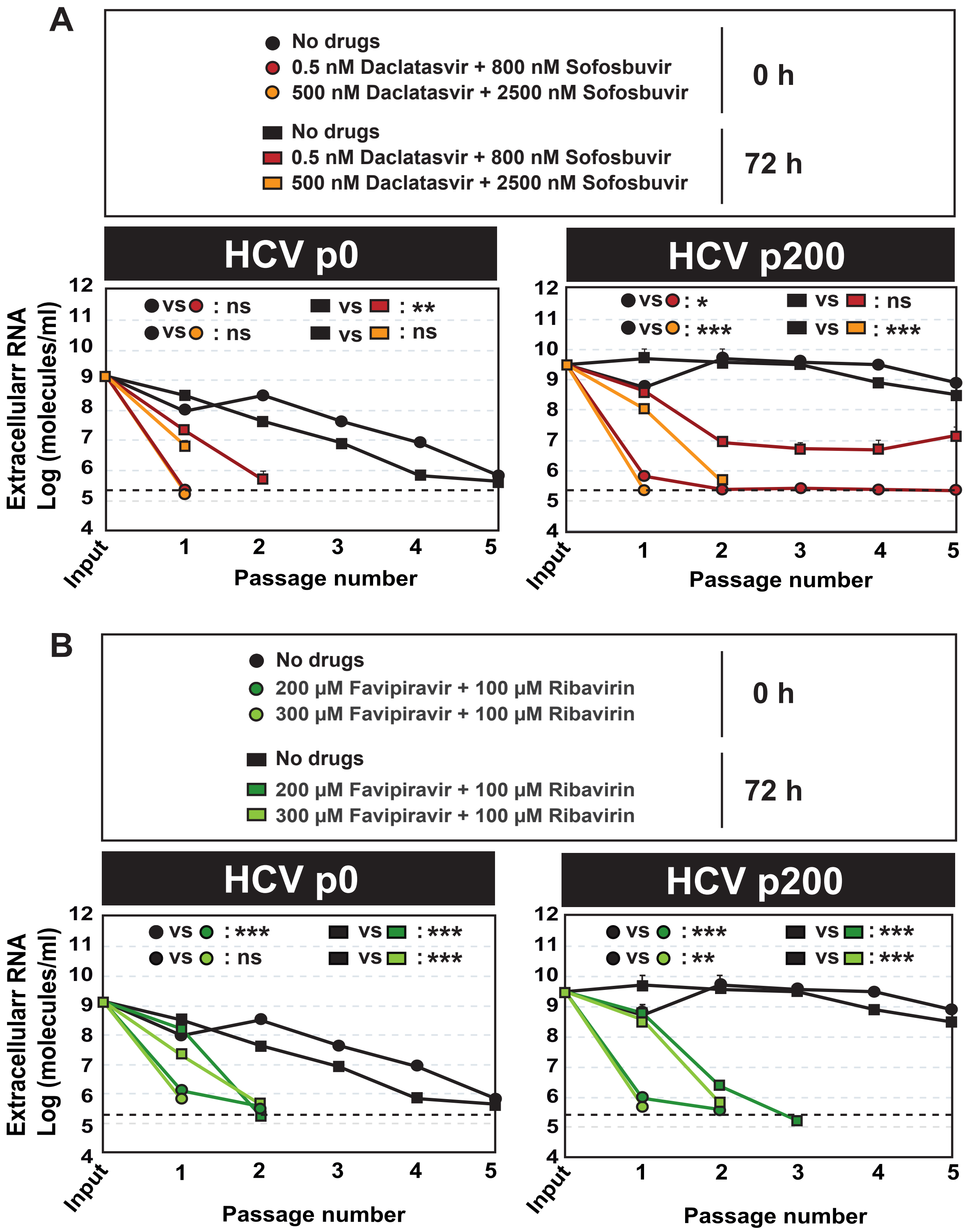

FIGURE S3. Extracellular RNA levels of HCV populations after serial passages in the presence of antiviral agent combinations. **(A)** The response of HCV p0 and HCV p200 subjected to 5 serial infections in the absence or presence of the Daclatasvir and Sofosbuvir indicated in the box. **(B)** The response of HCV p0 and HCV p200 subjected to 5 serial infections in the absence or presence of the Favipiravir and Ribavirin indicated in the box. The initial and the viruses at passage 1 are those described in Figure 3. The time of infection and of addition of the antiviral inhibitors is indicated; in all cases, extracellular RNA was extracted from cell culture supernatants and RNA was quantified at 72 h after addition of the inhibitors. For A, B, the statistical significance of the differences between values given in the bars are given as follows: ns: not-significant; * $P<0.05$; ** $P<0.01$; *** $P<0.001$; ANCOVA-test. Extracellular RNA values can be found in **Supplemental Table S5**.
